# Divergent Gemycircularvirus in HIV-Positive Blood, France

**DOI:** 10.3201/eid2111.150486

**Published:** 2015-11

**Authors:** Rathviro Uch, Pierre-Edouard Fournier, Catherine Robert, Caroline Blanc-Tailleur, Vital Galicher, Romain Barre, François Jordier, Philippe de Micco, Didier Raoult, Philippe Biagini

**Affiliations:** Centre National de la Recherche Scientifique, Marseilles (R. Uch, P.-E. Fournier, C. Robert, C. Blanc-Tailleur, V. Galicher, R. Barre, F. Jordier, P. de Micco, D. Raoult, P. Biagini);; Institut Hospitalo-Universitaire Méditerranée-Infection, Marseille, France (R. Uch, P.-E. Fournier, C. Robert, C. Blanc-Tailleur, R. Barre, F. Jordier, P. de Micco, D. Raoult, P. Biagini);; Aix-Marseille Université, Marseille (R. Uch, P.-E. Fournier, C. Robert, C. Blanc-Tailleur, V. Galicher, R. Barre, F. Jordier, P. de Micco, D. Raoult, P. Biagini);; Unité Mixte de Recherche 7268 ADES, Marseilles (R. Uch, V. Galicher, R. Barre, F. Jordier, P. de Micco, P. Biagini);; Etablissement Français du Sang, Marseilles (R. Uch, V. Galicher, R. Barre, F. Jordier, P. de Micco, P. Biagini);; Unité de Recherche sur les Maladies Infectieuses et Tropicales Emergentes, Marseilles (P.-E. Fournier, C. Robert, C. Blanc-Tailleur, D. Raoult)

**Keywords:** gemycircularvirus, HIV, blood, viruses, France

 **To the Editor:** Gemycircularviruses are a group of recently discovered single-stranded DNA viruses, found initially in fungi in 2010 (*1*). These “myco-like” viruses have a genome ranging from 2.1 to 2.3 kb, containing 2 opposite open reading frames that probably code for a capsid protein (CP) and a spliced replication-associated protein (Rep). Related viruses have been subsequently identified in animal blood and fecal matter, raw and treated sewage, and insects and plant material, suggesting that gemycircularviruses may represent a large group of viruses exhibiting considerable genetic diversity (*2*–*8*). The presence of these viruses was recently extended to humans after gemycircularvirus sequences were identified in human blood and brain tissue (multiple sclerosis patient), cerebrospinal fluid, and fecal matter (*8**,**9*).

While investigating the virome content of an HIV-positive blood donation, we identified several gemycircularvirus-related sequences. The initial metagenomic approach involved an HIV-1–positive plasma sample (B genotype, ≈530 copies/mL) obtained from the French blood agency national plasma bank in Tours, France. A 4-mL aliquot was prepared for metagenomic analysis after filtration, concentration, and nucleases treatment. Next, particle-protected nucleic acids were recovered and used for the preparation of a next-generation sequencing library and its subsequent analysis (Technical Appendix). Gemycircularvirus sequences identified among reads (1,680 vs. 82,560 reads total; ≈2%) were assembled into a resulting full-length sequence (GemyC1c) by using CodonCode Aligner version 5.1 (CodonCode Corporation, Centerville, MA, USA). This sequence was verified by using back-to-back specific primers, and the amplicon was cloned and sequenced according to the Sanger method.

The analysis of the GemyC1c sequence (2,109 nt, GenBank accession no. KP987887) revealed a genome divergent from those already available in databases, despite a similar genomic organization (Figure, panel A) and assignment to gemycircularviruses after BLAST (http://blast.ncbi.nlm.nih.gov/Blast.cgi) analysis of putative CP and Rep proteins. This divergence was demonstrated by the phylogenetic analysis of the deduced CP (Figure, panel B), which exhibited ≈72% and ≈44% aa pairwise identity with the 2 closest gemycircularvirus CP sequences available in GenBank (gemycircularvirus c from mongoose feces [Conceicao-Neto N., unpub. data] and HCBI8.215 from cattle blood, respectively). Moreover, GemyC1c CP exhibited ≈30% pairwise identity with viral sequences identified previously in humans (BZ1 from feces, SL1 from cerebrospinal fluid, MSSI2.225 from blood). The deduced spliced Rep (major Rep1 and minor Rep2), seen in such viruses, contained putative rolling circle motifs I (LFTYS), II (HLHAFVD), and III (YATKD) retrieved from gemycircularviruses (*4*).

**Figure:**
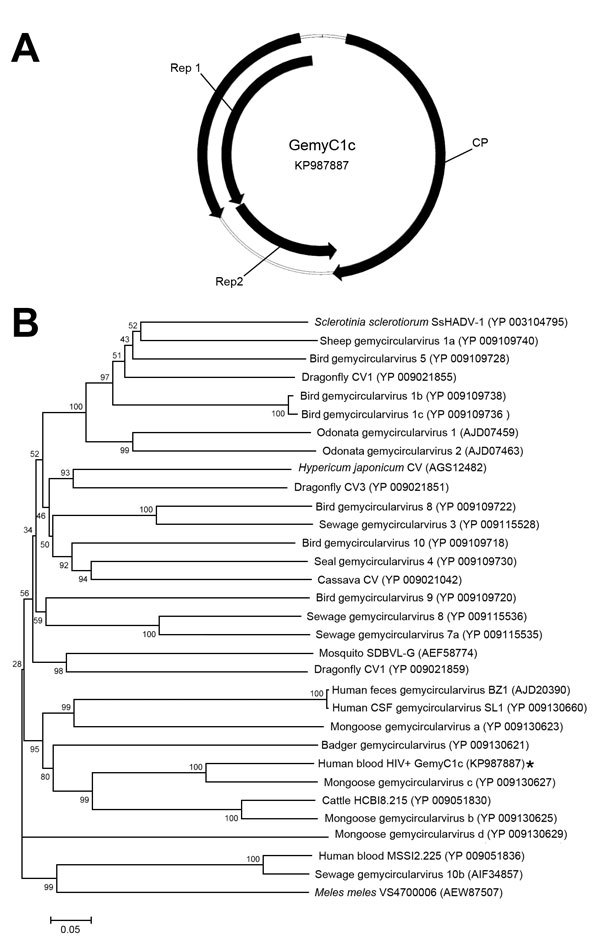
A) Predicted genomic structure of divergent gemycircularvirus (GemyC1c) isolated from HIV-positive blood, France. Arrows represent major open reading frames. Deduced capsid protein (CP) and spliced replication-associated proteins (Rep1/Rep2) are composed of 318 and 226/126 aa, respectively. B) Neighbor-joining phylogenetic tree constructed by using CP amino acid sequences of GemyC1c (asterisk) and genetically related gemycircularviruses. Bootstrap values were based on 1,000 replicates. Scale bar indicates amino acid substitutions per position.

We subsequently investigated the presence of GemyC1c DNA in 128 HIV-positive plasma samples (French blood agency national plasma bank) along with 256 HIV-negative plasma samples (healthy blood donors, southeastern France; mean donor age 38 years; 136 men; 1 man:1.13 women). Plasma samples were prepared as described previously (*10*), and extracted nucleic acids were tested for GemyC1c DNA by using a specific PCR that included negative, positive, and extraction controls (Technical Appendix).

Application of the above GemyC1c DNA detection system did not generate any positive signal in the 384 plasma samples in this study, suggesting that the presence of this virus in the blood of the populations tested was a rare occurrence. However, it is possible that other, divergent, GemyC1c-related sequences could be present in human blood but remain undetectable by the molecular assay used; the development of universal gemycircularvirus PCR systems is now expected.

Gemycircularviruses are potentially very stable in the environment. Because an unknown part of this group is able to infect fungi, possible contamination from the laboratory environment or nucleic acid extraction methods must be considered. The fact that the same genome was never identified in other libraries generated in our laboratory supports the absence of local contamination; of note, we were also able to detect GemyC1c DNA with PCR by using another plasma aliquot extracted by an alternative method (NucliSENS magnetic extraction; bioMérieux, Marcy l’Étoile, France) In addition, the systematic elimination of the first 35 mL of each blood donation, associated with filtration procedures and control of the temperature of stored plasma (−25°C), contributes to the reduction of bacterial/fungal contamination during blood collection. However, it is not possible to state that the GemyC1c sequence would belong to a human-tropic virus because an association of the virus with an unknown fungus is plausible. Thus, the presence of fungi in the gut, with fungi/virions having traversed the gut lining, or circulating in blood should be considered. Such aspects should prompt future investigations of the effective replication of gemycircularviruses in human or other mammalian cells.

Our discovery of the GemyC1c by a sequence-independent molecular approach was informative for several reasons: 1) this viral sequence would have been undetectable by PCR according to the high genetic divergence existing between GemyC1c and other gemycircularviruses identified; 2) this finding adds clues to the identification of potential new co-infections occurring in HIV-infected persons; and 3) this finding underlines the need to investigate the virome content of blood samples in a research context of new microbes as potential threats for transfusion. Further studies aimed at exploring genetic diversity and natural history of gemycircularviruses in human hosts are needed.

**Technical Appendix.**  Additional methods used to detect gemycircularvirus in an HIV-positive blood donation and sequence of the detected virus (GemyC1c).
