# Supplementary material for: Divergent Gemycircularvirus in HIV-Positive Blood, France
Source: Emerg Infect Dis. 2015 Nov;21(11):2096–8. doi: 10.3201/eid2111.150486 (PMC4622245; doi:10.3201/eid2111.150486)
Supplement: Supplementary file 1 — Technical Appendix. Additional methods used to detect gemycircularvirus in an HIV-positive blood donation and sequence of the detected virus (GemyC1c). [file 15-0486-Techapp-s1.pdf]

Article DOI: http://dx.doi.org/10.3201/eid2111.150486

Divergent Gemycircularvirus in HIV-Positive Blood, France

Technical Appendix

Plasma sample preparation and NGS analysis

A 4-ml aliquot of the plasma sample was clarified by centrifugation, filtered (0.22 µm), and viral particles were concentrated by adding sterile polyethylene glycol (MW 8,000, Invitrogen, Cergy Pontoise, France) to a 4% final concentration before centrifugation (10,000 × g for 1 h at 4°C). The pellet was suspended in 600 µl of sterile water, treated with 550 U of Benzonase (Sigma, Saint-Quentin Fallavier, France), 300 U of DNase and 1.2 mg of RNase (both from Roche Diagnostics, Meylan, France), and incubated at 37°C for 45 min. Particle-protected nucleic acids were recovered using the High pure viral nucleic acids kit (Roche) and subsequently used for the preparation of a NGS library according to manufacturer's instructions (Nextera XT DNA sample preparation kit; Illumina, Paris, France); the final analysis was performed on a MiSeq sequencer (Illumina).

GemyC1c DNA specific detection and controls

Nucleic acids were tested for GemyC1c DNA by using a nested-PCR assay targeting the CP gene with primers C1cS1 (5′-GTTCCGATTTCCACCGATTTCCA-3′) / C1cR1 (5′-TGCAACTCAACAGTTTCTTGGAG-3′) for PCR-1 (206 bp), and primers C1cS2 (5′-GATTTCCAACAACAGAATCCTGG-3′) / C1cR2 (5′- CTTGGAGGCCTTTGACGAAACAC-3′) for PCR-2 (175 bp). Amplification conditions for the two consecutive PCR assays were 94°C for 3 min, followed by 40 cycles of 94°C for 30 s, 58°C for 40 s and 72°C for 1 min. Dilutions of a cloned partial sequence of GemyC1c permitted to estimate the sensitivity of the amplification assay to be <5 copies of target sequence by limiting-dilution assay. Negative (sterile water) and positive controls (cloned GemyC1c partial sequence) were used systematically. The quality of

the nucleic acids extraction procedure was also checked by a control PCR targeting members of the family *Anelloviridae*, a highly prevalent group of DNA viruses in humans (*1*).

GemyC1c sequence (2,109 nt, GenBank accession no KP987887)

```
   1 taatattacc tctggacctt ggacccggac ccaggccggg tataaatagg tcccttcccc
  61 cgcggagatg tttttttttg ttgaggggta tgtcgcattt tacgtcatgg cttacgcaca
 121 ccgtcgcttt cggaggcgca ggttcgcccg ccgccggcca acattcgccc gtcgccgtgt
 181 cccccgtcgc tcttatggtc gccgtcgtcg gtcatccggt cgttccgttc ggtccattcg
 241 gaacatagcg gctcgcaagt gtcgcgacaa catgcttggc gttccgattt ccaccgattt
 301 ccaacaacag aatcctggcc ctctcaccat gtttggtgat accagccatg gatatttgtt
 361 ttctcccact gctcgtcgcg tcggttctct tgatgggtca ttacctcgcg gtattcacgc
 421 caattccgct gagcgttgga agacccggtg tttcgtcaaa ggcctccaag aaactgttga
 481 gttgcagcct aataatggag caggctggct gtggagacgc gtcgtatttt cctgcattgg
 541 cctattggag gaattcccta ccaatagtgt ggctgcgccc gactccactc gtgggtatgg
 601 tcgcgccatt tggaacatgc gggatggtag cgctgctgcc acccccccttt acaacgaaat
 661 ggccaattat cttttcgagg gtactcgatt gcgtgattgg atcaatcctt ttactgcgaa
 721 gttggatcgc agtattgtta cggtacattc ggacacgaca cgtaccattc agagtaacaa
 781 cccgaacggt acctacaaga ttttcaagcg gtattacccc gtgaacaggg gtattgtcta
 841 tgcggatgac gagtctggaa gtgggtcgaa gaatgacgcc cactacgctg ctggtacgac
 901 caaagggaat tcgggcgatc tgtttgtttt ggacctcttc aatgctatca atgatagttc
 961 ggagaatact atgaatttcg gagttcacgc caggtattac tggcatgagg gctctggaga
1021 gtaaagcctc tcccctatat aaactataat acaatttttt tccaaccaat catggtcgac
1081 gtcctcgctc attgggtcat tgttggatag ccaaatgcat ggtttacccc atgcgatgtg
1141 cttcttcttc ctgtacctgt cagtacagta gaactctgtc tgttgaccca accagctctt
1201 gtatgctggg aagaatttga aacctccttg gatgtcgtcg aatactgcat acgaacagtc
1261 tggatcctgt tcatactctt ccaaagagaa taatccacca aagtaggcat gggtgccaaa
1321 tgacctcgcc aacattgtct taccagtctt tgaaggaccc cacagataca agctcattcc
1381 acgcacctct gtcactcaga ggttagccca attagggtta gggttagggt tagggttagg
1441 gttagggtta gggttagggt tacccttagg gttagcggag tacttaccaa tctgattgcc
1501 ggatagatgt tccctaaccc attcccgaat ctcaccatat tgtgtaagat cgaacgagta
1561 ttggctaggg tgacgataag gtgcccgatc cactctaaac ttccaatctg cgtatgcaga
1621 gaatgaagtg aatgatttga cgagatggct gaaaagatgc gctgacgcaa gttcaaaaaa
1681 ctcctctcga gttgatgcat ctctgagttg gtcccatcca gcattctgtc gttcagcatt
1741 agaaccctgg ctaggttctg gtctgcgtag gctcccgcca catatgtcgc catctttcgt
1801 tgcgtaatcc caactgccac ttggacttcg gattgtagaa atgatatttg gatggaaacc
1861 ctcaacatcg aatttgcgca cgtctcgagt tctgtacttt ctgcgaaaat cgacgaaagc
1921 atgtagatgc gtcccaccat cgccgtgcgc ttctctgccg acgatacaat ctgctgagag
1981 agccgagaat aactccacga ctgctgatgg tcggagttgg ccgcattgcg agtaggtgaa
2041 taatccatag cgggcttgga atcgaaatga tgacataagc tttttcggaa gggtctggtc
2101 cagagtttt
```

Location:

CP: 68..1024; Rep1/Rep2: complement 997...2076; Overprinted ORF: complement 1392..2048.

Reference

1. Biagini P, Touinssi M, Galicher V, de Micco P. KIs virus and blood donors, France. Emerg Infect Dis. 2012;18:1374–5. PubMed http://dx.doi.org/10.3201/eid1808.120442
